# Ecological Compensation Standard of Trans-Boundary River Basin Based on Ecological Spillover Value: A Case Study for the Lancang–Mekong River Basin

**DOI:** 10.3390/ijerph18031251

**Published:** 2021-01-30

**Authors:** Yue Zhao, Feng-ping Wu, Fang Li, Xiang-nan Chen, Xia Xu, Zhi-ying Shao

**Affiliations:** 1 School of Business, Hohai University, Nanjing 211100, China; zh_yyyyy@hhu.edu.cn (Y.Z.); lf@hhu.edu.cn (F.L.); 180213120002@hhu.edu.cn (X.-n.C.); 170212070004@hhu.edu.cn (X.X.); shao_zy@hhu.edu.cn (Z.-y.S.); 2 National Engineering Research Center of Water Resources Efficient Utilization and Engineering Safety, Nanjing 210098, China

**Keywords:** ecological spillover value, trans-boundary river basin, ecological compensation standard, emergy-water resources ecological footprint model, Lancang–Mekong River Basin

## Abstract

Ecological compensation is an effective means to solve the conflict of interests among trans-boundary river basin countries. How to determine the ecological compensation standard is the core of ecological compensation. On the basis of the emergy synthesis method, we developed an emergy-water resources ecological footprint model for trans-boundary river basin countries. Based on the calculation of ecosystem service value and consumption ecological value of trans-boundary river basin countries, the ecological spillover value of each basin country is obtained. From the perspective of supply and consumption, the ecological compensation standard in basin countries is determined by judging the supply and consumption status of ecological services and combining with the willingness to pay for ecological compensation. Taking the Lancang–Mekong River Basin as an example, the results show that (1) the ecosystem service value of the Lancang–Mekong River Basin countries from high to low is Laos, Cambodia, Thailand, China, Vietnam, and Myanmar; (2) in terms of ecosystem service value consumption, the order from high to low is Thailand, Cambodia, Vietnam, China, Laos, and Myanmar; and (3) Thailand and Vietnam, located in the lower reaches of the basin, belong to the consumers of ecological services, and based on the actual willingness to pay, they need to pay $46.913 billion and $1.699 billion, respectively.

## 1. Introduction

River Basin is a complex region with natural geography and socio-economic attributes and functions connected by water resources. According to Trans-boundary Freshwater Dispute Database (TFDD), at present, there are 310 trans-boundary river basins in the world, involving 150 countries and regions [1]. With the development of international society and economy, the complexity of political relations and the global shortage of fresh water resources, the competition among trans-boundary river basin countries on the utilization of water resources is becoming increasingly severe. Moreover, the imbalance between the utilization of water resources and the protection of ecological environment is becoming more prominent, which leads to the imbalance of interests between upstream and downstream countries. Due to the mobility and geographical characteristics of trans-boundary rivers, in order to maintain the stability of the ecological environment of trans-boundary rivers, the upstream countries are limited in the development and utilization of trans-boundary water resources. They pay more efforts in protecting the ecological environment, thus bearing more protection costs and opportunity costs, while the downstream countries enjoy more ecosystem service value and benefits brought by the basin. It is easy to cause conflicts among basin countries. So how to coordinate the interest relations among basin countries, reduce the occurrence of conflicts, maintain the ecological security of trans-boundary rivers, and promote the sustainable development of water resources has caused more and more discussion.

As an economic means to coordinate the interests of stakeholders, ecological compensation can effectively solve the problem of interest conflicts among countries in trans-boundary river basins. The determination of ecological compensation standard is the core of ecological compensation activities [2], which is related to whether the ecological compensation activities can be carried out smoothly and how the compensation effect is. The number and range of compensation standards are affected by the loss or income of stakeholders in trans-boundary river basin, compensation time, national relations, and other factors. Due to the different factors and perspectives considered by different people when setting the compensation standards, there is no unified calculation method for compensation standards.

How to establish a scientific and reasonable ecological compensation standard of trans-boundary river basin has become the main focus and a difficult issue of current studies. At present, the existing standard formulation methods mainly include protection cost method, willingness to pay method, and ecosystem service value method: (1) The protection cost method, which includes direct cost method and opportunity cost method. Li Fen et al. [3] used the direct cost method of ecological protection to determine the ecological compensation standard in Sanjiangyuan area. Pham et al. [4] believed that the compensation standard of optimal efficiency should be determined according to the opportunity cost of the services provided. Zhang Tao [5] used opportunity cost method to calculate the ecological compensation standard of Xijiang River Basin. (2) The willingness to pay method. Willingness to pay is the level of compensation that can be accepted by the subject and object of compensation. In the calculation of compensation standard, the willingness to pay is considered to ensure the acceptance and recognition of relevant stakeholders. Plantinga et al. [6], Nyongesa et al. [7], Fan Hui et al. [8], Zhou Chen [9] et al. investigated residents’ willingness to pay through questionnaires and interviews respectively, and determined the ecological compensation standard according to this. (3) The ecosystem service value method. Since Costanza quantitatively measured the ecosystem service value in 1997 [10], more and more scholars set the ecological compensation standard according to the ecosystem service value. Zeng Xiangang et al. [11], Liu Lihua et al. [12], Gao Xin et al. [13] took ecosystem service value, net value of ecosystem service, and change value of ecosystem service as the basis for determining ecological compensation standard, respectively.

To sum up, scholars have reached an agreement on the determination of river basin ecological compensation standards, and carried out a series of related studies. Although the research perspectives and research methods are different, the ultimate goal of scholars is to formulate a set of feasible ecological compensation standard scheme. From the current research trend of river basin ecological compensation standard, in the selection of research methods, ecosystem service value method is more widely used to determine the ecological compensation standard, compared with the other two methods. At present, many scholars have combined it with other methods, which provides scientific support for the determination of river basin ecological compensation standard.

However, due to the huge value of ecosystem services, the ecological compensation standard based on the ecosystem services value is too high, and the operability is low in reality. Therefore, we consider that only after deducting the ecosystem service value of its own consumption, namely, the ecological spillover value, can it be scientific as an ecological compensation standard.

Therefore, how to accurately calculate the ecological spillover value is of great significance for the determination of ecological compensation standard. However, there are relatively few researches on ecological spillover value now, most of which are aimed at the measurement of ecosystem service value. At present, the calculation of ecosystem service value mainly includes material quality method and value measurement method [14]: (1) The material quality method, which mainly includes equivalent factor method. On the basis of Costanza, Xie Gaodi [15] constructed the unit area service value table of China’s terrestrial ecosystem, and clearly gave the value of different ecological services in the ecosystem. (2) The value measurement method, which mainly includes market value method, opportunity cost method and contingent valuation method [16]. Using the market value method, Colin M. Beier et al. [17] estimated that the loss value of broad-leaved forest affected by acid rain was about 10,000 US dollars. Using the opportunity cost method, Mesfin Tilahun et al. [18] inferred that the opportunity cost of protecting Ankasha forest reserve was US $92.66/ha to US $692.42/ha. Using the contingent valuation method, Zhang Cainan et al. [19] estimated the ecosystem cultural service value of Qilian Mountain National Park.

Nevertheless, the material quality method and value measurement method have some shortcomings, such as strong subjectivity [20], inconsistent evaluation standards [21], and easy to produce errors [22]. Especially in trans-boundary river basins, due to the large differences in economic, social, political, and ecological development levels among different countries and regions, it is particularly important to measure the ecosystem value fairly and reasonably. Emergy synthesis method is a method to convert different types of non-comparable materials and energy in the ecosystem into a comparable scale based on solar energy [23]. This method solves the problem of different evaluation criteria of ecological service value, and has been well used in the practice of measuring ecological service value. Elliott T. C. et al. used the emergy synthesis method to measure the economic value of forest ecosystem services in Maryland [24]. Wang Yiqi and Li Guoping calculated the emergy of ecosystem services in the upper reaches of Weihe River Basin based on the emergy synthesis method, and then extended it to determine the ecological compensation standard [25]. Wang Xianjin and Zhong Changbiao estimated the emergy of ecosystem service value of coastal beach reclamation area in different periods by using the emergy model [26].

The emergy synthesis method can accurately measure the ecosystem service value in different trans-boundary river basin countries. In order to express the ecological spillover value, it is necessary to consider their own consumption on this basis. The water resources ecological footprint method is a common method to measure the regional water consumption in a certain period of time [27]. Combining the water resources ecological footprint method with the emergy synthesis method, it can effectively measure the ecological spillover value of different trans-boundary river basin countries.

Based on the analysis, this study selects the Lancang–Mekong River Basin (LMRB) as the study area, constructs the emergy-water resources ecological footprint model, calculates the ecological spillover value (ESV) of the trans-boundary river basin (TBRB), and formulates the ecological compensation standard (ECS) of the TBRB according to the ESV. The purpose of this study is to promote the sustainable utilization of water resources and enhance the water ecological security of TBRBs. The main contribution of this study is to construct a relatively fair and objective emergy-water resources ecological footprint model to measure the ESV, and to formulate the ECS for TBRBs.

**Figure 1:**
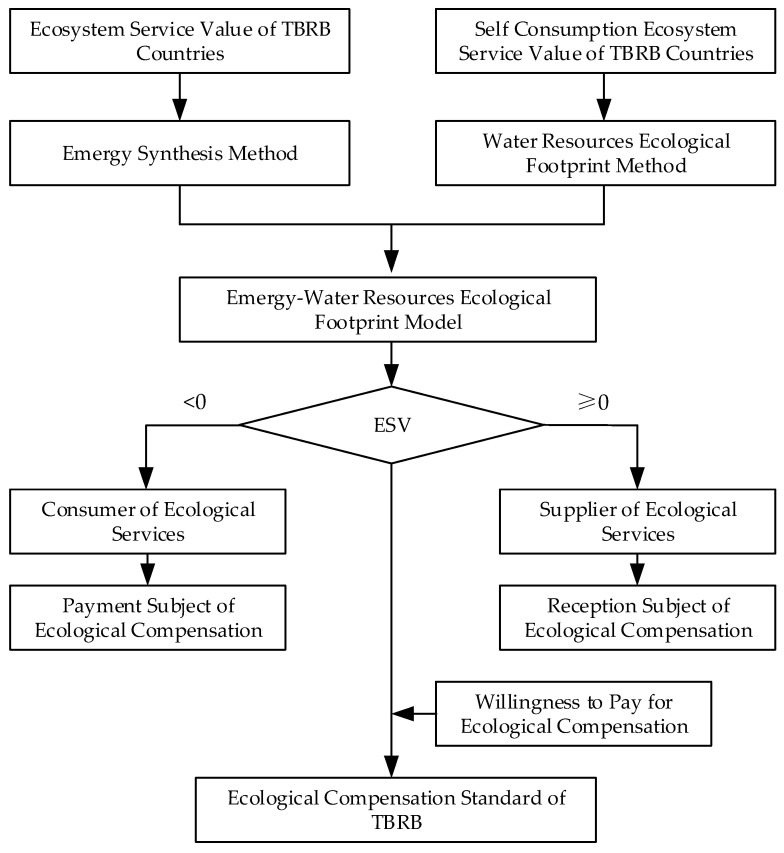
Logical structure of ecological compensation standard determination in trans-boundary river basins.

The rest of this paper is structured as follows: the second section introduces the study area and the data sources; the third section describes the research methods; the fourth section analyzes and discusses the relevant results; and the fifth section is the main conclusion of this paper. An outline is shown in Figure 1.

## 2. Study Area and Data Sources

### 2.1. Study Area

**Figure 2:**
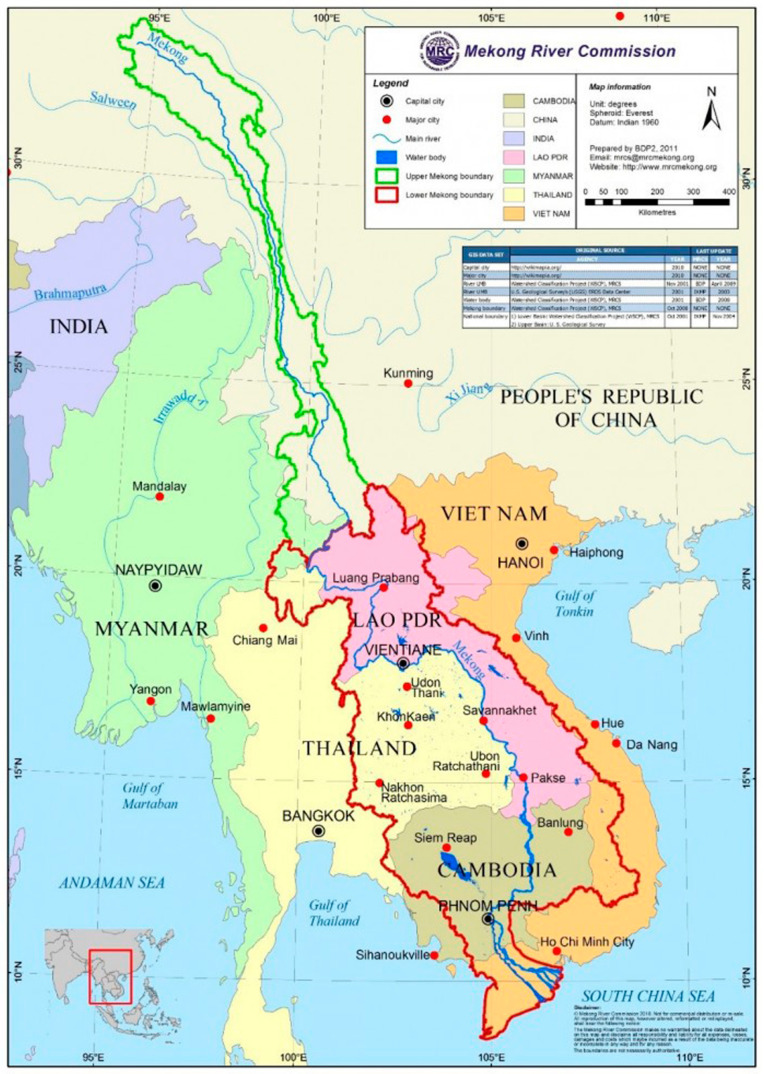
Geographical location of the Lancang–Mekong River Basin (LMRB) [31].

Lancang–Mekong River is an important trans-boundary river in Asia. It originates from the Qinghai–Tibet Plateau of China and flows through Myanmar, Laos, Thailand, Cambodia, and Vietnam, with a total length of 4884 km. The total basin area is 8.10 × 10^5^ km^2^, in which the proportions of China, Myanmar, Laos, Thailand, Cambodia, and Vietnam are 20.59%, 2.59%, 26.51%, 22.44%, 19.85%, and 8.02%, respectively [28]. The region is rich in natural resources. Water and hydropower resources are abundant, and rainfall is sufficient in rainy season. The average annual precipitation of China, Myanmar, Laos, Thailand, Cambodia, and Vietnam are 1.517 m/a, 1.747 m/a, 1.649 m/a, 1.733 m/a, 1.379 m/a, and 1.828 m/a, respectively [29]. There are many exchanges between basin countries in terms of transportation, trade, and so on. However, there are constant contradictions among basin countries in the development, utilization and protection of water resources in the basin. For example, the water transfer plan in Northeast Thailand has aroused opposition from Vietnam and other countries on water allocation. Laos’ construction of hydropower stations on the main stream has triggered disputes with Thailand, Vietnam, Cambodia, and other countries [30]. The conflicts and disputes between the countries in LMRB due to the unbalanced interest demands, affect the water security and sustainable development of the basin. It is appropriate to coordinate the interests among countries through the ecological compensation scheme. The study area is listed in Figure 2.

### 2.2. Data Sources

The data of runoff, basin area, population, GDP, and related water consumption of the six countries in the LMRB are from TWAP database [32], TFDD [33], Mekong River Commission [31], World Bank [34], and existing literature [30,35,36]. Solar radiation and rainfall are obtained according to Food and Agriculture Organization of the United Nations (FAO) Database [29]. The Engel’s coefficient comes from the Economic Research Bureau of the U.S. Department of Agriculture [37]. Detailed indicators are shown in Table 1.

## 3. Methods and Models

### 3.1. Theoretical Interpretation of Taking the ESV as the ECS

In order to determine the ECS for TBRBs, the connotation of ecological compensation should be clarified. At present, it is generally believed that ecological compensation is a kind of activity to charge for the behavior of damaging the environment and to compensate for the behavior of protecting the environment [38,39,40,41]. It involves the supply and consumption subjects of ecological environment or ecological services. The supply subject can carry out ecological protection well, while the consumption subject has caused a certain degree of damage to the ecological environment. In theory, the consumer is responsible for the compensation, and the supplier is compensated.

When determining the amount of compensation for the damaged ecological environment or ecological services, Habitat Equivalency Analysis (HEA) [42] provides us with a way of thinking; that is, the damaged ecological services and compensated ecological services should achieve a one-to-one balance. In the TBRB, the residents of the basin consume the ecological value of the basin in their daily life. When the consumption exceeds the ecological value of the basin itself, it will cause certain damage to the ecological environment of the basin and need to be compensated. From the perspective of supply and consumption, the compensation amount of the consumer should be equal to that of the supplier. Therefore, this paper puts forward the idea of taking the ESV as the theoretical value of ECS.

The ESV studied in this paper refers to the residual value of ecosystem service value of TBRB after deducting the ecological value consumed by residents in the basin. Due to the different geographical location and economic and social development level of each basin country in TBRB, the ESV of different regions is different, which can produce positive or negative spillover on the production and life of residents in other regions. On the one hand, when there is positive ESV, there is a surplus in the ecological service value of the region. This part of the residual value can not only reflect the self-development value of the region to protect the sustainable development of the overall ecological environment of the basin, but also can be used for the development of other regions. It belongs to the main body of the ecological service supply and should obtain ecological compensation funds. On the other hand, when the negative ESV occurs, there is a deficit in the value of ecological services in the region, which indicates that the region has overused the value of ecological services in the process of its own development, causing a certain degree of damage to the ecological environment of the basin. It belongs to the main body of ecological service consumption and should pay ecological compensation funds. It can be found that the ESV of the TBRB in a certain period corresponds to the amount of ecological protection or damage in that period, which provides a basis for this paper to take the ESV as the theoretical value of ECS of TBRB.

### 3.2. Determination of ESV

Based on the analysis of Section 3.1, the measurement of ESV of TBRB should include two parts: one is the estimation of total ecosystem service value (TESV) of TBRB, the other is the calculation of consumption of ecosystem service value (CESV) in basin countries. In the process of calculation, the rationality, scientificity, and comparability of ECS should be fully reflected. In this study, when formulating the ECS for TBRBs, it is reasonable to eliminate the CESV on the basis of TESV, and use the ESV as the ECS. In the selection of methods, the emergy method is used as the basic method to expand, combining the ecological footprint and the ecological carrying capacity of water resources. The emergy of ESV can be obtained by deducting the CESV, which can be converted by the emergy-currency ratio. It is scientific and comparable to reflect the ecosystem service value of the objective existence and consumption of TBRBs in the form of emergy.

On the basis of the above analysis, the emergy-water resources ecological footprint model of ESV is constructed, which is used as the basis for determining the theoretical value of ECS for TBRB. This can be expressed as Equations (1) and (2):

(1) $${\mathrm{ESV}}_{i}{=\mathrm{SE}}_{i}\cdot \frac{1}{\beta }$$

(2) $${\mathrm{SE}}_{i}={\mathrm{FE}}_{i}-{\mathrm{CE}}_{i}$$

where i, i = 1, 2, …, n is the basin country (BC) of TBRB; ${\mathrm{ESV}}_{i}$ is the ESV of each BC in TBRB, and the unit is $; ${\mathrm{SE}}_{i}$ is the emergy of ESV of each BC, and the unit is sej; β is the emergy-currency ratio of countries in TBRB; that is to say, the ratio of emergy for TESV to GDP of BCs, and the unit is sej/$; ${\mathrm{FE}}_{i}$ is the emergy of TESV in different BCs, and the unit is sej; and ${\mathrm{CE}}_{i}$ is the emergy of CESV for each BC, and the unit is sej.

#### 3.2.1. Determination of Emergy of TESV in BCs

We chose energy analysis method to calculate the TESV of the TBRB, mainly considering the environmental input energy of the ecosystem, including solar energy, wind energy, chemical energy of rainwater, and chemical energy of river [43]. By converting different energy inputs into energy values in the form of solar energy values, the emergy of TESV for the TBRB can be obtained, that is, the emergy embodiment of the service value that the TBRB ecosystem can provide for human production and life. According to the principle of emergy synthesis, the emergy model of TESV for TBRB was constructed, as shown in Equation (3):

(3) $${\mathrm{FE}}_{i}=\overset{4}{\underset{j=1}{\sum }}{\mathrm{EE}}_{\mathrm{ij}}\cdot {\mathrm{TR}}_{j}$$

$$\{\begin{array}{c} {\mathrm{EE}}_{i1}={\mathrm{DA}}_{i}\cdot \theta_{1}\\ {\mathrm{EE}}_{i2}={\mathrm{DA}}_{i}\cdot {\mathrm{WH}}_{i}\cdot \theta_{2}\cdot \theta_{3}\cdot \theta_{4}\\ {\mathrm{EE}}_{i3}={\mathrm{DA}}_{i}\cdot {\mathrm{AR}}_{i}\cdot \theta_{5}\\ {\mathrm{EE}}_{i4}=R_{i}\cdot \theta_{6}\cdot \theta_{5} \end{array}$$

where i, i = 1, 2, …, n is the BC of TBRB, j, j = 1, 2, 3, 4 represents the solar energy, wind energy, chemical energy of rainwater and chemical energy of river respectively; ${\mathrm{EE}}_{\mathrm{ij}}$ is the energy values corresponding to different natural energy inputs, and the unit is sej; ${\mathrm{TR}}_{j}$ is the emergy conversion rate representing different natural energy inputs, and the unit is sei/J. The specific index parameters are shown in Table 2.

#### 3.2.2. Determination of Emergy of CESV in BCs

River basin is an independent, complete and self-contained natural catchment unit, which is the spatial existence form of water resources in the ecosystem. The core of a river basin is water resources. The ecological value consumption of BCs in the TBRB is mainly the consumption of water resources in the basin. Therefore, this study calculated the CESV of BCs by calculating the water resources consumption coefficient (WRCC). WRCC is the ratio of water resources consumption to water supply in each BC. Combined with the emergy of TESV in BCs, the emergy of CESV in BCs is obtained, as shown in Equation (4):

(4) $${\mathrm{CE}}_{i}={\mathrm{FE}}_{i}\cdot \frac{{\mathrm{WD}}_{i}}{{\mathrm{WS}}_{i}}$$

where ${\mathrm{WD}}_{i}$ is the water resources consumption by each BC, and the unit is m^3^; ${\mathrm{WS}}_{i}$ is the water resources supply of each BC, and the unit is m^3^; $\frac{{\mathrm{WD}}_{i}}{{\mathrm{WS}}_{i}}$ is the WRCC. When $0<\frac{{\mathrm{WD}}_{i}}{{\mathrm{WS}}_{i}}<1$, it means that the consumption of water resources in each BC is less than the supply of water resources, and the water resources are in a sustainable state. When $\frac{{\mathrm{WD}}_{i}}{{\mathrm{WS}}_{i}}>1$, it means that the consumption of water resources in each basin is greater than the supply of water resources, and the water resources are in an unsustainable state. With the increase of $\frac{{\mathrm{WD}}_{i}}{{\mathrm{WS}}_{i}}$ value, the sustainable utilization degree of water resources gradually decreases.

In order to better measure the level of sustainable use of water resources in BCs, the WRCC is used to measure the pressure intensity of social and economic systems on regional water resources by referring to the research of Zhao Xiangui et al. [44], so as to reflect the ecological security state of regional water resources. The specific classification criteria are shown in Table 3.

- The water resources consumption by each BC

It can be expressed by the ecological footprint of water resources, which shows the utilization of water resources by various BCs in the form of water area [45]. The utilization of water resources includes agricultural water, industrial water, domestic water and ecological water used by BCs in the trans-boundary rivers. The calculation of ecological footprint of water resources can be expressed as Equation (5):

(5) $${\mathrm{WD}}_{i}{=N}_{i}\cdot \gamma_{w}\cdot (W_{i}/P_{w})$$

where $N_{i}$ is the total population of each BC, and the unit is person; $\gamma_{w}$ is the global equilibrium factor of water resources; $W_{i}$ is the water consumption per capita in each BC, and the unit is m^3^; $P_{w}$ is the global average production capacity of water resources, and the unit is m^3^/hm^2^; the other symbols are the same as above.

- The water resources supply of each BC

It can be expressed through the water resource ecological carrying capacity, indicating the maximum support that water resources can provide to the production, living, and ecology of the BCs in a specific stage of development in order to meet the sustainable development of the basin. It is generally believed that a country or region should deduct at least 60% of its water resources for the maintenance of ecological environment [46]. The calculation of water resource ecological carrying capacity can be expressed as Equation (6):

(6) $${\mathrm{WS}}_{i}=1-60\%\cdot \gamma_{w}\cdot \varphi_{i}\cdot Q_{i}/P_{w}$$

where $\varphi_{i}$ is the yield factors of water resources in each BC; $Q_{i}$ is the total amount of water resources in each BC, and the unit is m^3^; the other symbols are the same as above. As far as $\varphi_{i}$ is concerned, it is the ratio between the average water resource production capacity of each BC and the global average water resource production capacity, as shown in Equation (7):

(7) $$\varphi_{i}=\frac{P_{i}}{P_{w}},\;P_{i}=\frac{V_{i}}{{\mathrm{DA}}_{i}}$$

where $P_{i}$ is the average water resource production capacity of each BC, and the unit is m^3^/hm^2^; $V_{i}$ is the total amount of water resources in each BC in the calculation period, and the unit is m^3^; and the other symbols are the same as above.

### 3.3. Construction of ECS Model for TBRB

According to the analysis in Section 3.2, the ${\mathrm{ESV}}_{i}$ of TBRB is the theoretical value of ECS for TBRB. However, Li Jinchang, Jiang Wenlai et al. believe that people’s attention to ecological environment value will gradually increase with the improvement of people’s living standards, showing a development trend of rapid rise, slow rise to saturation, similar to the change trend of S-shaped growth curve [47]. Therefore, they proposed to combine the S-shaped pearl growth curve model with the Engel’s coefficient representing the level of economic and social development and people’s living standards to obtain the relative value of the willingness to pay for the ecological environment value. They used it and the effect was remarkable. Since then, scholars have been using the model to calculate the adjustment coefficient of actual payment for ecological value (ACAPEV) [48]. It can be expressed as Equation (8):

(8) $$l_{i}=\frac{1}{{1+e}^{{-t}_{i}}},\;t_{i}=\frac{1}{E_{i}^{k}}-3$$

where $l_{i}$ is the ACAPEV, which is the increasing function of $t_{i}$, ranging from 0 to 1. Its function image is called the Peel Growth Curve. $E_{i}^{k}$ is the Engel’s coefficient of each BC in k year.

Referring to the research of Li Jinchang et al., the actual value of ECS is obtained based on the willingness to pay for ecological value of each BC. It can be expressed as Equation (9):

(9) $${\mathrm{CS}}_{i}=l_{i}\cdot {\mathrm{ESV}}_{i}$$

where ${\mathrm{CS}}_{i}$ is the actual value of ECS of each BC, and the unit is $; the other symbols are the same as above.

## 4. Results and Discussion

### 4.1. Emergy of TESV in LMRB

Since the functional expression of the conversion of ecosystem inputs into solar energy in TBRB has been determined, according to Equation (3), the emergy of TESV in LMRB countries can be calculated (${\mathrm{FE}}_{i}$), as shown in Table 4:

As the Table 4 shows, the total emergy of TESV in LMRB was 11.48 × 10^25^ sej, ranging from high to low were Laos, Cambodia, Thailand, China, Vietnam, and Myanmar. Among them, the emergy of TESV in Laos was the highest. This is mainly because its basin area and runoff are in the first place among the six countries in the basin, and its superior natural conditions make it get more natural inputs among the six countries in the basin, so it has higher emergy of TESV. The difference of basin area and runoff among Cambodia, Thailand and China is not significant, which explains that the emergy of TESV of these three countries is also similar. In Myanmar, the emergy of TESV only accounts for 2.00% of the whole basin due to its lowest basin area and runoff.

### 4.2. Emergy of CESV in LMRB

#### 4.2.1. The Ecological Footprint of Water Resources in LMRB

According to Equation (5) and the study of Huang Linnan et al. [49], the value of $\gamma_{w}$ was 5.19 and $P_{w}$ was 3140 m^3^/hm^2^. The calculated results of the ecological footprint of water resources in LMRB (${\mathrm{WD}}_{i}$) were shown in Table 5:

It can be seen from Table 5 that, in terms of the total ecological footprint of water resources, the BCs were Thailand, Vietnam, Cambodia, China, Laos, and Myanmar from high to low. The ecological footprint of water resources reflects the consumption degree of water resources in the BCs. According to the results, the consumption of water resources from Lancang–Mekong River in Thailand, Vietnam, and Cambodia is much higher than that in Laos, China, and Myanmar.

Geographically, Laos, Thailand, Vietnam, and Cambodia are located in the lower reaches of the Lancang–Mekong River, while China and Myanmar are located in the upper reaches. Except Laos, the consumption of water resources in the downstream countries is significantly higher than that in the upstream countries, which is related to the development and utilization mode of water resources in BCs. The upstream countries mainly use non-consumable water models such as hydropower and shipping, while the downstream countries mainly use consumable water models, and irrigation, fishery, and so on are developed [35]. For Laos, from the perspective of Mekong River, it is located in the upper reaches of Mekong River. The main way of water use is hydropower development, which belongs to non-consumable water use. The water consumption of Laos in the basin is only 2.2% of its own water production, and 16.08 × 10^10^ m^3^ water flows out of the country every year [50].

#### 4.2.2. The Ecological Carrying Capacity of Water Resources in LMRB

According to Equations (6) and (7), the calculated results of the ecological carrying capacity of water resources in LMRB (${\mathrm{WS}}_{i}$) were shown in Table 6.

It can be seen from Table 6 that among the six countries in LMRB, the water resources ecological carrying capacity of Laos was significantly higher than that of other countries in the basin, which was 287.91 × 10^6^ hm^2^ and took a leading position in the river basin. There was no significant difference between Cambodia, Vietnam, Thailand, and China in the ecological carrying capacity of water resources, which were 110.51× 10^6^ hm^2^, 88.77× 10^6^ hm^2^, 74.58 × 10^6^ hm^2^, and 73.96 × 10^6^ hm^2^, respectively. Compared with other BCs, Myanmar had a low water resources ecological carrying capacity, which was only 7.85 × 10^6^ hm^2^. It was 36.68 times lower than that of Laos, which was closely related to the difference of runoff between the two countries.

#### 4.2.3. The Emergy of CESV in LMRB

According to Equation (4), combined with the emergy of TESV, ecological footprint of water resources and water ecological carrying capacity of each BC in LMRB, the WRCC, and the emergy of CESV (${\mathrm{CE}}_{i}$) can be calculated. The specific results were shown in Table 7:

It can be seen from Table 7 that Thailand had the highest consumption of ecological value among the countries in the basin, which was 25.55 × 10^24^ sej. Its WRCC was 1.30, indicating that Thailand had been in a state of imbalance between supply and demand in the utilization of water resources of Lancang–Mekong River, which was not conducive to the sustainable development of water resources. Except Thailand, the WRCC in Vietnam was 1.09, showing the imbalance of supply and demand. The existing water resources can no longer meet its own development needs. The other four countries in the basin, China, Myanmar, Laos, and Cambodia, all had the WRCC less than 1.0, and the imbalance between supply and demand had not yet occurred. However, the WRCC in Cambodia was 0.85, and the utilization degree of water resources was relatively high, indicating that there were some hidden risks in the utilization of water resources. Therefore, it is worthwhile for local water resources management departments to think deeply about whether the existing water resources utilization mode can be carried out for a long time.

In order to better measure the level of sustainable use of water resources in BCs, the WRCC is used to measure the pressure intensity of social and economic systems on regional water resources. According to the contents in Table 3 and Table 7, it could be concluded that the water resource ecological security levels of China, Myanmar, Laos, Thailand, Cambodia, and Vietnam were very safe, very safe, very safe, relatively unsafe, slightly unsafe, and relatively unsafe, respectively. There were different levels of ecological security of water resources in different BCs.

Among them, China, Myanmar, and Laos were in a very safe state, indicating that the utilization of water resources of the Lancang–Mekong River by these three countries was relatively reasonable. They effectively maintained the ecological health and stability of the river, and could realize the sustainable development of the river.

Cambodia’s water ecological security level was relatively low compared with the three countries mentioned above. Although it has not yet appeared the state of imbalance between supply and demand, it was in a slightly unsafe state, indicating that it had a high degree of development and utilization of rivers, which had a certain impact on the sustainable development of rivers. In order to promote the long-term and stable development of rivers, it is necessary to strengthen the management of water resources and improve the utilization efficiency of water resources.

The ecological security of water resources in Thailand and Vietnam has shown a relatively unsafe state, indicating that their exploitation and utilization of water resources from Lancang–Mekong River has exceeded the load capacity of the Lancang–Mekong River within their own territory, which was not conducive to the sustainable development of the Lancang–Mekong River as a whole. Thailand’s use of the Lancang–Mekong River is mainly in agricultural irrigation and domestic water. In order to meet its own water needs, Thailand has formulated the Mekong river diversion plan for many times, introducing a large amount of Mekong river water into its own territory, which has caused a certain impact on the water consumption of downstream countries and caused dissatisfaction of downstream countries [51]. Vietnam has a large demand for irrigation water and ecological water. The lower Mekong Delta is an important granary base for Vietnam, where high-quality rice is grown and 90% of Vietnam’s rice exports are produced, so water consumption is high. For these two countries, domestic industrial structure should be adjusted to use water more efficiently and waste less water.

### 4.3. Emergy of ESV in LMRB

According to the calculation results of the emergy of TESV and CESV for BCs in LMRB, the emergy of ESV for each BC can be obtained, which were 1.68 × 10^25^ sej, 0.21 × 10^25^ sej, 3.93 × 10^25^ sej, −0.59 × 10^25^ sej, 0.32 × 10^25^ sej and −0.12 × 10^25^ sej for China, Myanmar, Laos, Thailand, Cambodia, and Vietnam, respectively.

It can be seen from the results that China, Myanmar, Laos, and Cambodia had positive ESV, which indicated that these four countries, after deducting their own consumption ecological value, could also provide ecological services to other countries in the basin. They were suppliers of ecological services and should receive ecological compensation funds. Thailand and Vietnam had negative ESV, indicating that these two countries had caused damage to the ecological environment of water resources in the basin in the process of their own development. They were consumers of ecological services and should pay ecological compensation funds.

In practice, China and Laos, located in the upstream, have invested a large amount of manpower and material resources in the construction of water conservancy projects in the basin, which can reduce the losses of downstream countries due to special water situation during the period of basin drought and flood. For example, China’s emergency water replenishment to Vietnam in 2016 effectively alleviated the drought in Vietnam. Therefore, according to the principle of benefit compensation, it is reasonable for countries that are consumers of ecological services to compensate countries that are suppliers of ecological services.

However, according to the results of Section 4.2.3, Cambodia’s water resources utilization was relatively high, which has been in a slightly unsafe state. The ecological environment of water resources in the basin is unstable, which cannot provide stable ESV for other basin countries. At present, it is urgent to consider how to maintain the stability of the ecological environment of water resources in the basin, so as to ensure the sustainable development of the basin without damaging the interests of other countries while maintaining the economic and social development of its own country. Therefore, we argue that Cambodia will neither receive nor pay compensation funds in this ecological compensation practice.

### 4.4. ECS of BCs

Based on the above analysis results, Thailand and Vietnam are consumers of ecological services. According to the principle of benefit compensation, from the perspective of the overall sustainable development of the river basin, these two countries should assume more responsibility for the ecological protection of the river basin and need to pay ecological compensation funds. Combining the emergy-currency ratio and the actual willingness to pay of Thailand and Vietnam, the actual value of ECS can be obtained according to Formulas (8) and (9), as shown in Table 8.

Known from Table 8, the theoretical value of ecological compensation amount to be paid by Thailand and Vietnam is 72.07 billion US dollars and 4.694 billion US dollars, respectively. In order to make the results more operable, the theoretical value of ecological compensation amount was adjusted in consideration of both countries’ economic development level and the willingness to pay for ecological compensation, so as to get the actual value of the final ecological compensation amount to be paid by both countries. In the end, Thailand and Vietnam should pay $46.913 billion and $1.699 billion, respectively, with Thailand paying the highest amount of actual compensation.

In this study, the establishment of ECS for TBRB provides a specific quantitative basis for the realization of TBRB ecological compensation mechanism. At the same time, it is conducive to guiding BCs to take initiatives to improve the utilization rate of water resources within their own borders, maintain the ecological health of TBRB, and promote the overall sustainable development of river basins. In terms of specific compensation model, we believe that it is not only limited to financial compensation, but also flexible negotiation can be conducted according to the interests of the basin countries through trade, water rights trading, engineering construction, political compensation and other approaches. In terms of the utilization of ecological compensation funds, we suggest that a Trans-boundary River Basin Ecological Protection Fund can be constructed, and the ecological compensation funds paid by ecological consumers can be used for the overall ecological protection of the basin. The Fund Committee is composed of all basin countries, aiming at promoting the sustainable development of the basin as a whole.

## 5. Conclusions

In order to reduce the conflicts of interest caused by water use in BCs of TBRB and realize the sustainable development of basins, we discussed the setting of ECS based on the ESV from the perspective of basin integration. First of all, we used emergy synthesis method to measure the TESV in TBRB. Then, we used the water resources ecological footprint method to measure the CESV in TBRB, and on this basis, we got the ESV of each BC. Finally, the final ECS was determined by judging the ecological surplus and ecological deficit status of the BCs and combining with the ecological compensation payment willingness of the BCs.

We took the LMRB as an example for empirical research, and the main conclusions are as follows:

(1) The TESV of BCs in LMRB from high to low is Laos, Cambodia, Thailand, China, Vietnam, and Myanmar. Among them, the TESV of Laos is the highest, accounting for 34.93% of the whole basin. Myanmar has the lowest TESV, only at 2.00% of the basin level.
(2) The CESV of BCs in LMRB from high to low is Thailand, Cambodia, Vietnam, China, Laos, and Myanmar. Among them, Thailand and Vietnam belong to the consumers of ecological services, causing damage to the ecological environment of water resources in the basin, and water resources present a relatively unsafe state. Cambodia, Laos, China, and Myanmar are suppliers of ecological services. Cambodia consumes more water resources in the basin than Laos, China, and Myanmar, showing a slightly unsafe state of water resources, while the remaining three countries are in a good state of water resources, showing a very safe state.
(3) According to the principle of benefit compensation, Thailand and Vietnam, located in the lower reaches of the basin, need to pay ecological compensation funds and assume more responsibilities for the ecological protection of the basin. Based on the actual payment willingness of the two countries, it is determined that the two countries need to pay US $46.913 billion and US $1.699 billion respectively, and Thailand needs to pay the highest amount of actual compensation.

The ecological compensation in TBRB involves multiple interests, which is more complex in the actual operation process. This paper provides an idea for the setting of ECS in TBRB. However, there are still some limitations in this study, especially due to the lack of basin information sharing mechanism and the sensitivity of relevant data, which fails to consider the variation trend of TESV and CESV in BCs, and fails to consider water quality factors when determining ECS. In the future, BCs should establish the concept of a community with a shared future for water, carry out long-term and effective basin cooperation, share information related to TBRB water resources, and jointly safeguard and promote the sustainable development of basins.

## Figures and Tables

**Table 1:** Related indicators of LMRB.

	China	Myanmar	Laos	Thailand	Cambodia	Vietnam
Basin area (10^4^ km^2^)	16.50	2.40	20.20	18.40	15.50	6.50
Average solar radiation (10^9^ J/m^2^∙a)	6.00	6.80	5.98	6.54	6.80	6.20
Average rainfall(m/a)	1.52	1.75	1.65	1.73	1.38	1.83
Runoff(10^10^ m^3^/a)	7.61	0.95	16.62	8.07	9.02	5.23
Agricultural water(10^8^ m^3^)	14.07	1.16	29.57	93.52	27.49	267.76
Industrial water(10^8^ m^3^)	3.58	0.03	0.12	0.94	0.13	0.44
Domestic water(10^8^ m^3^)	1.91	0.07	1.16	9.35	1.27	4.43
Population (10^6^ person)	7.81	0.57	6.60	30.41	14.91	7.45
GDP (10^10^ $)	6.06	0.08	0.63	23.74	1.81	5.08
Engel’s Coefficient	0.241	0.566	0.605	0.276	0.446	0.411

**Table 2:** Emergy index parameters of trans-boundary river basin (TBRB) ecosystem.

Emergy	Calculation of Energy Inputs				Emergy Conversion Rate		
	Index	Symbol	Value	Unit	Symbol	Value	Unit
Solar energy	Basin area	${\mathrm{DA}}_{i}$	—	m^2^	$TR_{1}$	1	sei/J
	Average solar radiation	$\theta_{1}$	—	J/m^2^∙a			
Wind energy	Basin area	${\mathrm{DA}}_{i}$	—	m^2^	$TR_{2}$	1496	sei/J
	Wind energy height	${\mathrm{WH}}_{i}$	10	m			
	Air density	$\theta_{2}$	1.23	kg/m^3^			
	Eddy diffusion coefficient	$\theta_{3}$	2.01	m^3^/s			
	Gradient of wind speed	$\theta_{4}$	3.154 × 10^7^	s/a			
Chemical energy of rainwater	Basin area	${\mathrm{DA}}_{i}$	—	m^2^	$TR_{3}$	18199	sei/J
	Average rainfall	${\mathrm{AR}}_{i}$	—	m/a			
	Gibbs free energy	$\theta_{5}$	4.94 × 10^6^	J/g × g/m^3^			
Chemical energy of river	Runoff	$R_{i}$	—	m^3^/a	$TR_{4}$	48459	sei/J
	Water density	$\theta_{6}$	1 × 10^3^	kg/m^3^			
	Gibbs free energy	$\theta_{5}$	4.94 × 10^6^	J/g × g/m^3^			

Note: the parameters and data related to energy input index and emergy conversion rate refer to *Emergy Analysis of Eco-economic System* [43], “—” means to be determined according to the actual value of each basin country (BC).

**Table 3:** Classification of ecological security of water resources.

Level	WRCC	Representation State
I	<0.50	Very safe
II	[0.50, 0.80)	Relatively safe
III	[0.80, 1.00)	Slightly unsafe
IV	[1.00, 1.50)	Relatively unsafe
V	[1.50, 2.00)	Very unsafe
VI	≥2.00	Extremely unsafe

**Table 4:** Emergy of total ecosystem service value (TESV) for countries in LMRB unit: sej.

	China	Myanmar	Laos	Thailand	Cambodia	Vietnam	Total
Solar energy	9.90 × 10^20^	1.63 × 10^20^	12.09 × 10^20^	12.03 × 10^20^	10.53 × 10^20^	4.03 × 10^20^	50.21 × 10^20^
Wind energy	1.92 × 10^23^	0.28 × 10^23^	2.36 × 10^23^	2.15 × 10^23^	1.81 × 10^23^	0.76 × 10^23^	8.52 × 10^23^
Chemical energy of rainwater	2.25 × 10^22^	0.38 × 10^22^	2.99 × 10^22^	2.87 × 10^22^	1.92 × 10^22^	1.07 × 10^22^	10.41 × 10^22^
Chemical energy of river	1.82 × 10^25^	0.23 × 10^25^	3.98 × 10^25^	1.93 × 10^25^	2.16 × 10^25^	1.25 × 10^25^	11.37 × 10^25^
Emergy of TESV	1.84 × 10^25^	0.23 × 10^25^	4.01 × 10^25^	1.96 × 10^25^	2.18 × 10^25^	1.26 × 10^25^	11.48 × 10^25^

**Table 5:** Ecological footprint of water resources in LMRB unit: 10^6^ hm^2.^

Country	China	Myanmar	Laos	Thailand	Cambodia	Vietnam
Total ecological footprint of water resources	6.29	0.59	6.04	97.22	94.22	96.98

**Table 6:** Ecological carrying capacity of water resources in LMRB unit: 10^6^ hm^2.^

Country	China	Myanmar	Laos	Thailand	Cambodia	Vietnam
Ecological carrying capacity of water resources	73.96	7.85	287.91	74.58	110.51	88.77

**Table 7:** Emergy of CESV in LMRB.

	WRCC	The Emergy of CESV/sej
China	0.09	1.57 × 10^24^
Myanmar	0.07	0.17 × 10^24^
Laos	0.02	0.84 × 10^24^
Thailand	1.30	25.55 × 10^24^
Cambodia	0.85	18.59 × 10^24^
Vietnam	1.09	13.76 × 10^24^

**Table 8:** Ecological compensation standard (ECS) for consumer countries of ecological services.

	Emergy of ESV/sej	Emergy-Currency Ratio/sej/$	Theoretical Value of ECS/$	ACAPEV	Actual Value of ECS/$
Thailand	0.59 × 10^25^	0.08 × 10^15^	720.70 × 10^8^	0.65	469.13 × 10^8^
Vietnam	0.12 × 10^25^	0.25 × 10^15^	46.94 × 10^8^	0.36	16.99 × 10^8^

## Data Availability

The data presented in this study are openly available in [Food and Agriculture Organization of the United Nations] at [http://www.fao.org/faostat/en/#data], reference number [29]; [Mekong River Commission] at [http://www.mrcmekong.org/news-and-events/events/mrc-secretariat-affirms-mekong-basin-size-length/], reference number [31]; [Transboundary Waters Assessment Programmer (TWAP) Database] at [http://twap-rivers.org/#home], reference number [32]; [Transboundary Freshwater Dispute Database (TFDD)] at [https://transboundarywaters.science.oregonstate.edu/content/data-and-datasets], reference number [33]; [World Bank] at [https://data.worldbank.org.cn/country], reference number [34]; [Economic Research Service for United States Department of Agriculture] at [https://www.ers.usda.gov/], reference number [37] and available in [Cooperation and Disputes in Water Resources Development of Lancang-Mekong River (1957–2016)], [Study on available water resource allocation and water benefit sharing in Lancang-Mekong River] and [Differences of Water Resources Utilization among 5 Riparian Countries in the Lancang-Mekong River Basin].

## Abbreviations

LMRB	Lancang–Mekong River Basin
ESV	Ecological spillover value
TBRB	Trans-boundary river basin
ECS	Ecological compensation standard
TESV	Total ecosystem service value
CESV	Consumption of ecosystem service value
BC	Basin country
WRCC	Water resources consumption coefficient
ACAPEV	Adjustment coefficient of actual payment for ecological value
